# Computational Prediction of Intronic microRNA Targets using Host Gene Expression Reveals Novel Regulatory Mechanisms

**DOI:** 10.1371/journal.pone.0019312

**Published:** 2011-06-09

**Authors:** M. Hossein Radfar, Willy Wong, Quaid Morris

**Affiliations:** 1 Department of Electrical and Computer Engineering, University of Toronto, Toronto, Ontario, Canada; 2 Institute of Biomaterials and Biomedical Engineering, University of Toronto, Toronto, Ontario, Canada; 3 The Donnelly Centre for Cellular and Biomolecular Research, University of Toronto, Toronto, Ontario, Canada; 4 Department of Molecular Genetics, University of Toronto, Toronto, Ontario, Canada; 5 Department of Computer Science, University of Toronto, Toronto, Ontario, Canada; 6 Banting and Best Department of Medical Research, University of Toronto, Toronto, Ontario, Canada; University of Sheffield, United Kingdom

## Abstract

Approximately half of known human miRNAs are located in the introns of protein coding genes. Some of these intronic miRNAs are only expressed when their host gene is and, as such, their steady state expression levels are highly correlated with those of the host gene's mRNA. Recently host gene expression levels have been used to predict the targets of intronic miRNAs by identifying other mRNAs that they have consistent negative correlation with. This is a potentially powerful approach because it allows a large number of expression profiling studies to be used but needs refinement because mRNAs can be targeted by multiple miRNAs and not all intronic miRNAs are co-expressed with their host genes.

Here we introduce InMiR, a new computational method that uses a linear-Gaussian model to predict the targets of intronic miRNAs based on the expression profiles of their host genes across a large number of datasets. Our method recovers nearly twice as many true positives at the same fixed false positive rate as a comparable method that only considers correlations. Through an analysis of 140 Affymetrix datasets from Gene Expression Omnibus, we build a network of 19,926 interactions among 57 intronic miRNAs and 3,864 targets. InMiR can also predict which host genes have expression profiles that are good surrogates for those of their intronic miRNAs. Host genes that InMiR predicts are bad surrogates contain significantly more miRNA target sites in their 3′ UTRs and are significantly more likely to have predicted Pol II and Pol III promoters in their introns.

We provide a dataset of 1,935 predicted mRNA targets for 22 intronic miRNAs. These prediction are supported both by sequence features and expression. By combining our results with previous reports, we distinguish three classes of intronic miRNAs: Those that are tightly regulated with their host gene; those that are likely to be expressed from the same promoter but whose host gene is highly regulated by miRNAs; and those likely to have independent promoters.

## Introduction

MicroRNAs (miRNAs) are a large family of small, non-coding endogenous RNAs that play critical roles in a wide range of normal and diseased-related biological processes [1]–[3] by post-transcriptionally repressing the expression of target genes. miRNAs repress gene expression by binding target mRNAs often in their 3′ UTR.

MicroRNAs recognize their targets through partially complementary, as such, they are particularly amenable to computational prediction of their target mRNA sequences [4]–[20] (for a recent review of these techniques see [21]). Substantial computational and experimental effort in this area has revealed a number of core predictive sequence features: strong base pairing between the 3′ UTR of mRNAs and the miRNA seed region [22], thermodynamic stability of binding sites [23], evolutionary conservation of binding sites (particularly the seed region) [7], [14], secondary structure accessibility [8], [11], [24]–[26], and dinucleotide composition of flanking sequence [14], [27]. For example, TargetScan [8] is a popular method that incorporates many of these features and regularly performs well in head-to-head comparisons (e.g., [28]). For a comprehensive review of sequence-based features see [29].

However, despite these efforts, recent reports claim that even the most accurate miRNA target prediction methods have false positive rates greater than 30% [28], [30] and the limited overlap of their predictions suggest that they also have high false negative rates [31]–[33].

One strategy to improve the accuracy and the sensitivity of target prediction methods is to search for inverse relationships between paired miRNA and mRNA expression levels. Although miRNA-mediated gene repression can occur through Argonaute-catalyzed mRNA cleavage or mRNA destabilization, or translational repression [34]–[40], as much as 84% of the resulting decrease in the protein product is due to miRNA-induced changes at the transcriptional level [41]. This miRNA-induced mRNA degradation leaves a signature that is inversely correlated with miRNA expression level on the steady-state mRNA levels of its targets [34], [42], [43]. This signature can be detected even when miRNAs also repress translation [37], [38]. However, detecting this signature is difficult simply by comparing expression profiles of a single miRNA and mRNAs [44] possibly because many mRNAs are regulated by multiple miRNAs [12], [32]. We have previously shown that allowing for multiple miRNA regulators of a given mRNA and Bayesian modeling of potential sources of variation can reveal this signature [12]. One way to predict the miRNA targets is to identify mRNA-miRNA pairs whose expression profiles show significant negative correlation in both human and mouse data [45]. However, these approaches require large amounts of paired miRNA and mRNA expression data. This paired data is rarely available because different assays need to applied to the same RNA sample, and until recently, miRNA expression levels were difficult to measure accurately.

Approximately half of mammalian miRNAs are in the introns of protein-coding genes, so it may be possible to predict the targets of some of these intronic miRNAs without having to measure their expression level. Indeed, many intronic miRNAs appear to lack their own promoters and are processed out of introns [46]–[57]. Estimates for the proportion of intronic miRNA whose expression profiles are significantly correlated with their host gene vary between 34% (25/74 [51]) and 71% (22/31 [50]). If these co-expression relationships can be detected without having to measure the miRNA expression, then host gene expression levels can be used as a surrogate for the miRNA levels when doing target prediction (c.f., [16]). There are substantial advantages to doing this. First, host gene expression levels are measured at the same time and on the same platform as the target gene expression levels, thus removing the need to model platform and laboratory-based effects. Also, there are hundreds of suitable Gene Expression Omnibus datasets for well-studied model organisms that can be used for target prediction, thus adding considerable statistical power to any target predictions.

However, not all host gene expression profiles are useful for predicting the targets of their intronic miRNAs. Some of these intronic miRNAs show evidence of having their own promoter [58]–[65]. For example, two independent studies found putative promoters for one-third of intronic miRNAs [58], [59]. Furthermore, host gene mRNAs may themselves be under post-transcriptional regulation by other miRNA. As such, it is important to distinguish host genes with expression profiles that are good surrogates for those of their intronic miRNAs from those that are not.

Here we propose a new method that both identifies intronic miRNAs whose host gene's expression provide good surrogates for their expression level as well as predicting the mRNA targets of these miRNAs. Our method takes as input a set of potential miRNA target sites based on sequence comparisons and then among these sites it identifies those likely to be functional sites based on the degree to which host gene's expression is predictive of down-regulation of the mRNA. When predicting regulators of a particular mRNA, we consider the combined effect of all of its potential regulators because most miRNAs are regulated by multiple miRNAs [12], [31], [32], [66], [67]. Our method can use any mRNA expression profiles, however, here we use 140 gene expression data series chosen for their size and their use of the same microarray platform. We distinguish between good and bad host gene surrogates based on the proportion of their hosted miRNA's potential targets that we predict to be functional. Host genes that we deem to be bad surrogates based on this test have more predicted Pol II/III promoters in their introns as well as more predicted miRNA binding sites in their 3′ UTRs.

## Results

We modeled the change of an mRNA's expression level in a sample by a linear combination of the host gene expression levels of a subset of the miRNAs with potential target sites in the 3′ UTR of the mRNA. We distinguished the functional and non-functional target sites by fitting this linear model to expression profiling data from a large number of studies and then examining the distributions of weights assigned each potential miRNA regulator.

This linear modeling approaches differs from previous ones [12], [66], [67] in a number of important aspects. First, we use host gene expression levels as surrogates for miRNA expression levels. Also, we predict functional and non-functional sites by integrating evidence from multiple profiling studies rather than a single study. This change allows us to employ a much simpler linear model for each individual dataset because we need not rely upon prior assumptions to detect statistical signals of regulation. The parameters of our model can be easily estimated using ordinary least squares linear regression. One final change is that we assume that the multiple miRNAs contribute additivity to the down-regulation of a given mRNA rather than multiplicatively. In other words, the decrease in expression level of the target is proportional to the expression level of miRNAs. As such, we do not log transform the mRNA expression profile applying our model to it. In the following, we describe our methodology and obtained results in detail.

### 1-Computing weights for putative miRNA regulators on individual datasets

Our linear model is as follows: Given 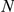 gene expression datasets 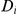, 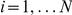 (see materials and Table S1), let 
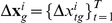
 denote an 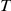-element vector whose elements correspond to the decrease in the expression level of the 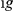
th target gene over 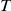 samples in the 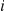
th dataset. We model this vector as a linear function of 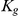 intronic miRNAs whose host gene expression levels are denoted by 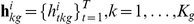. These intronic miRNAs represent putative regulators of the mRNA identified based on a sequence-based miRNA prediction algorithm, such as TargetScan. Based on the above assumptions and definitions, we build the following model:
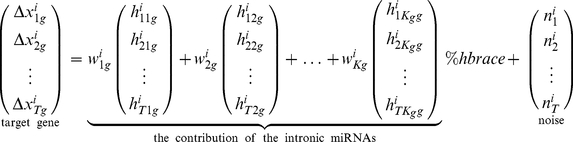
(1)where 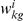, 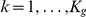 is a weight that represents the contribution of the 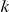
th intronic miRNA in regulating the target gene 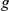 and 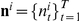 represents modeling error or noise. Typically, we cannot measure 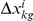 directly, so we approximate it by the difference between the mean mRNA expression level in the sample and the measured level of 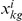, i.e., 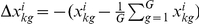 , where 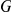 denotes the number of genes in the dataset. We also assume that the noise vector is sampled from a multivariate Gaussian distribution whose covariance matrix is proportional to the identity matrix, i.e., is spherical. Equation (1) can be written in matrix-vector notation as
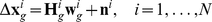
(2)in which 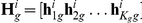 denotes the expression data of 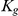 host genes over 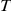 samples.

In the model, a positive (negative) weight, 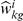, indicates the contribution of the host gene 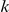 in decreasing (increasing) the expression level (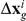) of the target gene 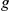. We call this the unconstrained linear model (ULM) to distinguish it from previous models [12], [66] that constrain the weights 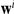 to be positive thereby insisting that miRNAs act only to down-regulate the expression of their target genes. We relax this constraint for convenience because doing so simplifies the fitting procedure without impacting the predictions of the model (see Fig. S2, Fig. S3, and Fig. S4). In this paper, we focus on the down-regulating role of miRNAs as only few miRNAs have been reported to up-regulate target gene expression [68], [69].

Under these assumptions, we can estimate 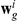 using ordinary least squares linear regression, i.e., we minimize the root mean squared error between the reconstruction of the mRNA down-regulation profile based on the miRNA estimates and the observed one, i.e.,:
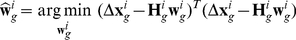
(3)where 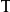 denotes the matrix transpose operation. Note that the solution to equation (3) corresponds to the maximum likelihood estimate of 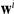 (see materials for details).

We solved (3) individually in each dataset to obtain 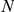 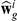 vectors for the target gene 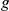. In order to be able to compare weights across datasets, we rescaled the weights for each mRNA within each dataset by dividing each element in 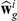 by the sum of the absolute values of its elements, i.e., 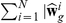 thus ensuring that 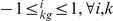. In the next section we describe how we combine weights from multiple datasets to make a single prediction for each putative miRNA and mRNA interaction. A summary of symbols used is given in Table 1.

**Table 1:** The description of symbols used in the paper.

symbol	Description
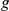	gene index
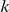	miRNA index
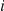	dataset index
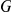	# of target genes
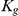	# of putative targeting miRNAs for gene 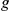
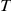	# of samples
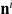	noise vector corresponding to dataset 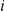
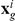	expression of gene 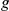 in dataset 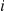
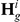	a matrix containing the expressions of host genes in dataset 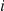
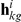	expression of the gene hosting miRNA 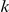 that targets gene 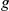 in dataset 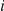
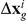	change in expression level of gene 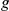 in dataset 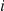
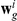	regulatory weights of miRNAs targeting gene 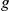 in dataset 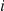

### 2-Mapping host gene weights to miRNA weights

**Figure 1:**
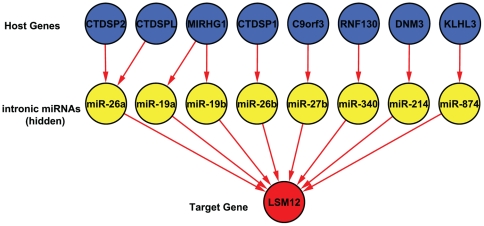
Interaction between hosts, targets, and intronic miRNAs using DAG. A directed acyclic graph (DAG) that represents interactions between host genes, intronic miRNAs, and the target. The top nodes represent the host genes. The middle layer represents the intronic miRNAs located in the introns of the host genes at the first layer. And the bottom layer denotes the target gene. In this DAG, the gene LSM12 is targeted by intronic miRNAs miR-19a, miR-19b,miR-26a,miR-26b, miR-27b, miR-214, miR-340, and miR-874 which are located in the introns of CTDSP2, CTDSPL, MIRHG1, CTDSP1, C9orf3, RNF130, DNM3, and KLHL3.

**Figure 2:**
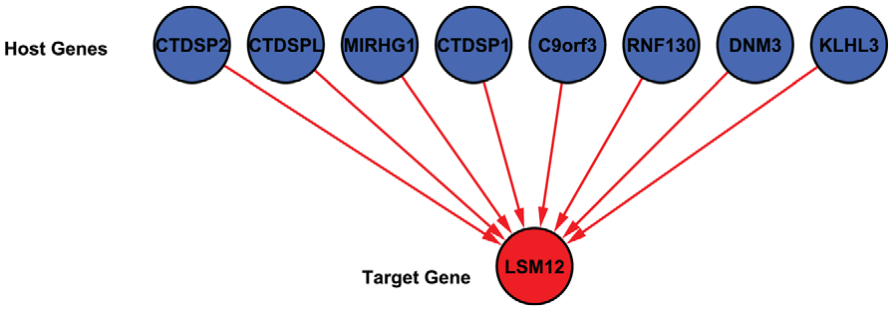
The simplified DAG. The simplified DAG of Fig. 1 in which host genes have a direct interaction with the target.

Our model uses host gene expression as a surrogate for the expression level(s) of its intronic miRNAs. This requires us to resolve some of the host gene / intronic miRNA relationships that are not one-to-one, because some host genes contain multiple intronic miRNAs and some intronic miRNAs are duplicated in more than one host gene. Fig. 1 shows a directed acyclic graph (DAG) representing these relationship for eight intronic miRNAs that are possible regulators for the expression of gene LSM12 whose protein product accumulates in stress granules [70]. This DAG can be interpreted as a graphical model in which the expression patterns of intronic miRNAs are hidden. Because our goal is not only to predict miRNA targets but also to determine which host genes are good surrogates for their intronic miRNAs, we assign weights directly to host genes rather than miRNAs. So, the host genes of duplicated miRNAs get separate weights. Also, when a host gene contains more than one intronic miRNA with putative targets in a given mRNA, we assign this host gene weight to each of these miRNAs. The host gene / target mRNA model that we fit for LSM12 after making these adjustments is shown in Fig. 2.

### 3-Combining multiple datasets to predict functional targets

We make our predictions of functional targets by comparing the distribution of weights assigned to a host gene / mRNA pair across the datasets to a distribution in which the association between host genes and their expression profiles is randomized. Specifically, we generate a null distribution of weights by permuting the labels of the host genes and re-calculating the weights for all putative pairs in every dataset. All of the weights calculated during this process comprise the empirical null distribution. Then for each host gene / mRNA pair, we compare the distribution of weights for this pair against this null distribution by calculating the two-sided Wilcoxon-Mann-Whitney (WMW) ranksum P-value, we call this value 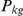 for the 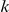-th host gene and the 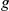-th mRNA. We also record whether the mean of the distribution of real weights for a given pair is larger or smaller than the mean of the null distribution. The means of the weight distributions that are larger than random reflect a prediction by our model that a miRNA associated with the host gene is down-regulating the target mRNA. As we will describe later, we use host gene / mRNA pairs whose weights are smaller than random when distinguishing good and bad host gene surrogates.

We interpret 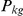 as an enrichment measure and determine a cutoff value, for both positive and negative enrichment, by comparing it to P-values calculated for host gene / mRNA pairs that are unlikely to interact. We generated P-values for these likely negative examples by calculating a two-tailed WMW P-value, 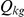, for each putative host gene / mRNA pair as described above except that we replace the actual weight distribution with that we computed after permuting the host gene labels. Formally, we define 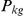 and 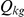 as follows:
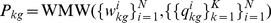
(4)




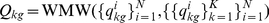
(5)where 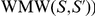 is a function that calculates a two-tailed WMW P-value for sets 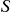 and 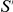 and 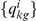 is the set of weights fit to the permuted data.

**Figure 3:**
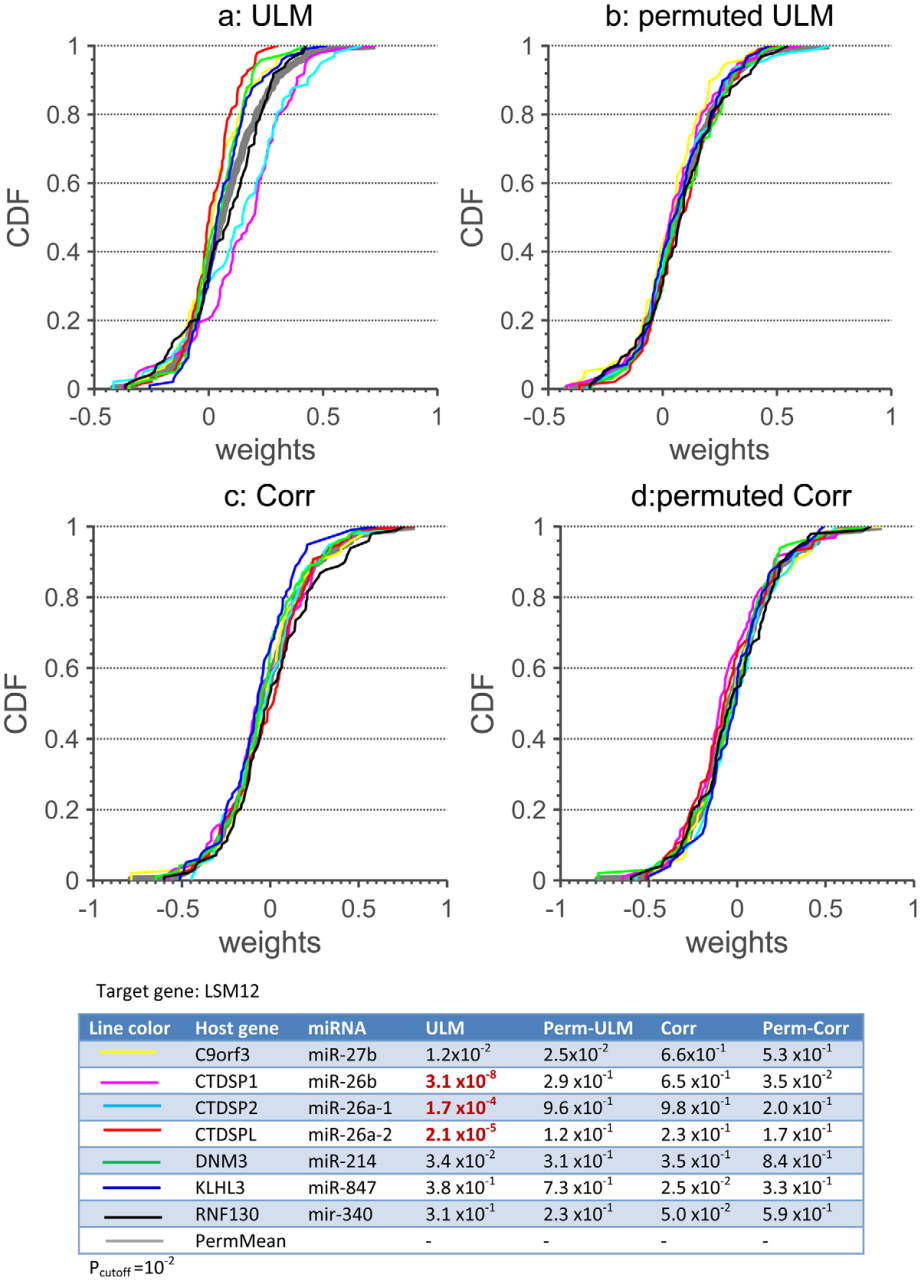
CDF plots for weights. Plots a–d: the CDFs of the weights 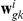 (a–b) and 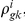 (c and d)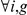 for seven host genes obtained from ULM (a and b), and CORR (c and d) with the actual (a and c) and permutation setups (b and d). The thick gray line in each plot is the CDF obtained from the pooled permutation data for each method. The Table lists the p-values (Willcoxon ranksum test) showing the probability that the weight or correlation data are drawn from the pooled permutated data (see (4) and (5) for detail). P-values marked in red are predicted to be significant (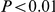). It should be noted that the host gene MIRHG1 was excluded for analysis since the expression data related this host gene did not exist in the retrieved dataset.

Fig. 3.a–d show the CDFs of weights (i.e. 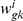 and 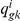 ,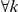) for all host genes whose intronic miRNAs have potential target sites in LSM12. The CDF of the pooled weights obtained from the permuted data (the thick gray line) is also shown. These weights were obtained from two methods: ULM (Fig. 3.a–b) and a method that sets weights by correlation (Fig. 3.c–d) (the CORR method, see materials for details). Recently, the HOCTAR method was introduced that uses inverse correlation with host genes to detect intronic miRNA targets [16]; here we use the CORR method to demonstrate how well inverse correlation performed within our framework. From Fig. 3.c–d, we see that the distributions obtained from CORR from the actual and permuted data are almost indistinguishable suggesting that CORR is unpowered and/or prone to misclassification compared to ULM. Moreover, these observations also confirm the cooperative impact of miRNAs on target genes. By contrast, the distributions of three host genes, namely CTDSP1,CTDSP2, and CTDSPL, obtained from ULM–also from constrained linear model (CLM) (Fig.S4)–are significantly different from their permuted counterparts and the pooled distribution. The table at the bottom of Fig. 3 lists 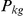 and 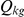 for each interaction. In the next subsection we specify a cutoff point in order to determine the significant interactions that we will be using to make predictions about targets.

### 4-Determining a cutoff value for significant interactions

**Figure 4:**
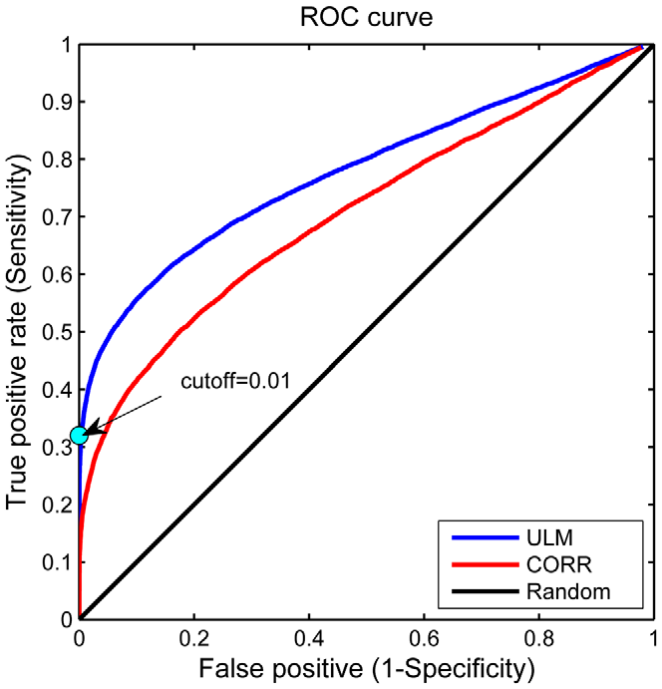
Receiver Operating Characteristic (ROC) curve analysis. Receiver Operating Characteristic (ROC) curve analysis to determine the cutoff point. We set the cutoff point to 0.01 (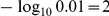) to identify significant host-target interactions. The blue, red, and black curves show the ROC associated with ULM, CORR, and random, respectively.

**Figure 5:**
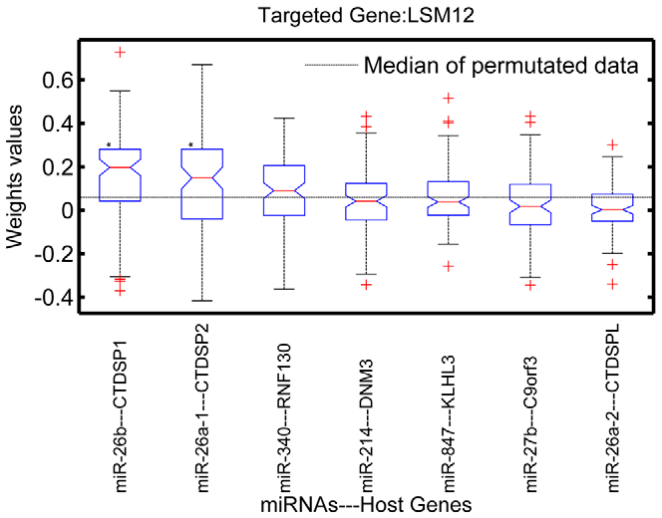
Interaction between LSM12 (target gene ) and the host genes of its targeting miRNAs. Shown are the boxplots of weights obtained from the procedure described Materials, Subsection 5. The significant negative interactions, i.e. those with 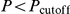 and 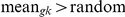, have asterisk marks. The horizontal dashed line indicates the median of weights obtained from the permutation test.

We apply ROC analysis to determine a cutoff point for specifying significant 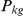. Fig. 4 shows the ROC curves for the ULM and CORR methods when we use 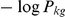 as the discriminant values for the positive examples and 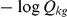 for the negative examples. By using a cutoff of 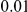 for the ULM 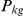 values, we are able to achieve a sensitivity of 32% at 100% predicted specificity. In other words, 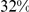 of interactions predicted by TargetScan are assigned weights whose distributions are more distinguishable from a random distribution than any of those assigned the permuted host gene / mRNA pairs. If we insist on 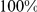 specificity, CORR only recovers 17% of the TargetScan predicted host gene / mRNA interactions; achieving 32% sensitivity with CORR requires lowering the specificity to 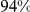. The corresponding cumulative distribution of these 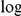 P-values is shown in Fig.S1-2. In the example in Fig. 3, detect significant interactions between CTDSP1 and LSM12 (P-value = 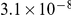
(ULM)), between CTDSP2 and LSM12 (P-value = 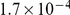 (ULM)), and between CTDSPL and LSM12 (P-values = 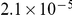 (ULM)) significant. Fig. 5 shows the boxplots of weights of 7 host genes whose intronic miRNAs putatively target LSM12.

### 5-Detecting good host gene surrogates

Using the method described in the last section, we defined for each host gene a set of significant interactions between the host gene's expression level and those of the predicted targets of its associated intronic miRNAs (i.e. those for which 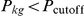). Furthermore, we know whether that an interaction is a “negative” one when the mean of weights over all datasets (i.e. 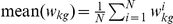) is larger than random expectation or a “non-negative” one, when the mean is smaller than random expectation. When we examine all the significant interactions between a host (or equivalently its miRNA) and its predictive targets, we find that these interactions are almost exclusively negative or non-negative.

**Figure 6:**
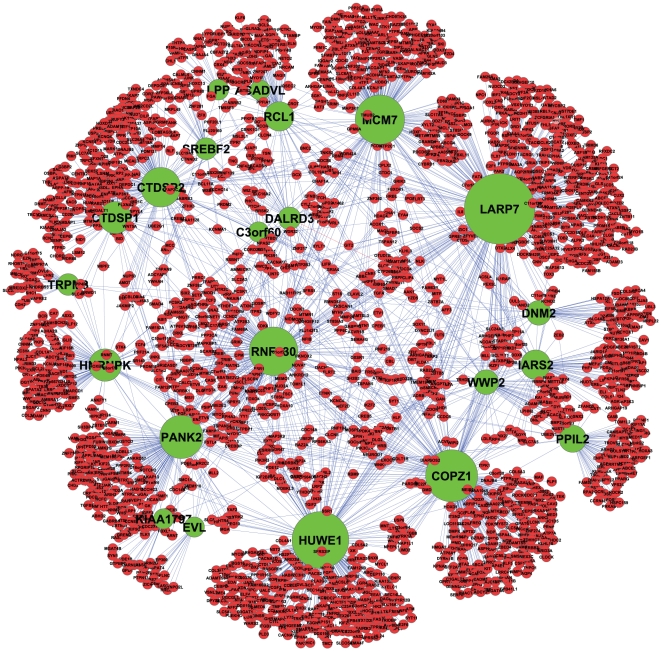
A gene-gene interaction network of target and host genes of intronic miRNAs. A gene-gene interaction network of target and host genes of intronic miRNAs with significant negative interactions. Each green and red node shows a host and target gene, respectively. An edge indicates that there is a significant negative interaction between two nodes, i.e. 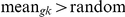 and 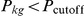. The size of each host node is proportional to the number of the edges connected to it. Host–intronic miRNAs pairs are: MCM7–miR-106b/93/25, LARP7–miR-367/302a/302b,LARP7–miR-302c/d, RNF130–miR-340,PPIL2–miR-130b/301b,HUWE1–miR-98/let-7f, CTDSP2–miR-26a, CTDSP1–miR-26b, RCL1–miR-101,COPZ1–miR-148b, PANK2–miR-103,TRPM3–miR-204, DNM2–miR-199a/638, IARS2–miR-215/194,HNRNPK–miR-7, SREBF2–miR-33a, WWP2–miR-140, DALRD3–miR-425/191, EVL–miR-342, LPP–miR-28, ACADVL–miR-324,KIAA1797–miR-491, C3orf60–miR-191.

We retrieved and processed the expression profiles of 75 host genes and 3864 target genes (see materials and Table S3 ) over 140 datasets. For all target genes (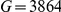), we carried out the procedure given in Materials subsection 5 for obtaining p-values for ULM, CLM, and CORR methods. All of these p-values are available in Table S3. We report the results for ULM, the significant interactions from CLM are similar and, as we described in the last section, using CORR reduces our sensitivity or specificity or both. After applying the cutoff at 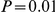, we find that 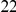 (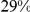) host genes have more negative interactions than positive ones. Those host genes and their 1935 target genes are shown in Fig. 6.

**Figure 7:**
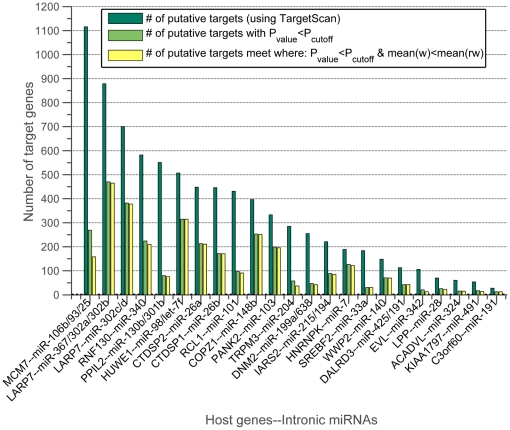
The host genes that significantly negatively interact with the target genes. Each dark green bar shows the number of putative targets–-obtained from TargetScan–-of intronic miRNAs of the corresponding host gene labeled in the x-axis. Light green bars indicate the number of putative targets which satisfy the condition 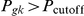 (significantly regulated). Number of putative targets that meet the both conditions 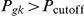 and 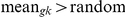 (significantly negatively regulated), are shown by yellow bars.

**Figure 8:**
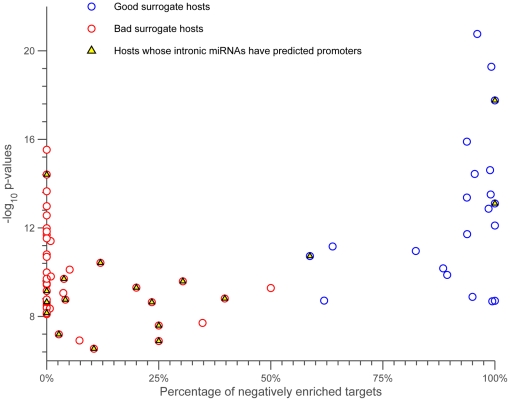
The scatter plot shows the enrichment of host genes. Each circle, associated with a host, shows the mean of 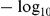 p-values of the enriched genes vs the percentage of negatively enriched genes targeted by the intronic miRNAs of host genes. The blue and red circles are associated with good and bad surrogate host genes, respectively. The circles corresponding to the hosts whose intronic miRNAs have predicted promoters marked by yellow triangles.

**Figure 9:**
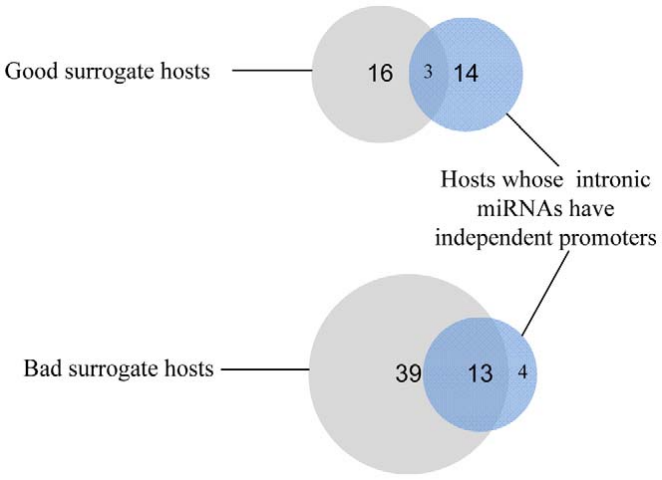
Venn diagrams. Venn diagrams showing overlap between good and bad surrogate host genes and hosts whose intronic miRNAs have predicted promoters.

Fig. 7 shows the number of TargetScan-predicted targets for each of these 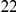 host genes, along with the number of significant interactions for these predicted targets and the number of these significant interactions that are negative. As shown, for 21 out of 22 host genes, almost all interactions are negative (equal light green and yellow bars). We take this as evidence that the host gene expression level is a good surrogate for that of its intronic miRNAs. Indeed when we consider all of the host genes with any significant interactions, we find that they fall into two main classes: those whose interactions are almost exclusively negative and those that are non-negative (Fig. 8). Furthermore, those that are non-negative are highly enriched for those with possible promoters, as predicted by sequence analysis in [58], for their intronic miRNAs (Fig. 8 and Fig. 9). We also observe that significantly negatively enriched host genes have, on average, high mean p-values (blue circles). For instance, 7 out of 8 host genes, namely HNRNPK , COPZ1, HUWE1, PANK2, ACADVL, LARP7, and IARS2 appear at the top of the ranked mean p-value list. Thus, significantly negatively interactions and high mean p-values are two determinants which may provide strong evidence for detecting co-expressed host-intronic miRNA pairs.

### 6-Targeting of host genes by miRNAs partially explains their predicted surrogacy

**Figure 10:**
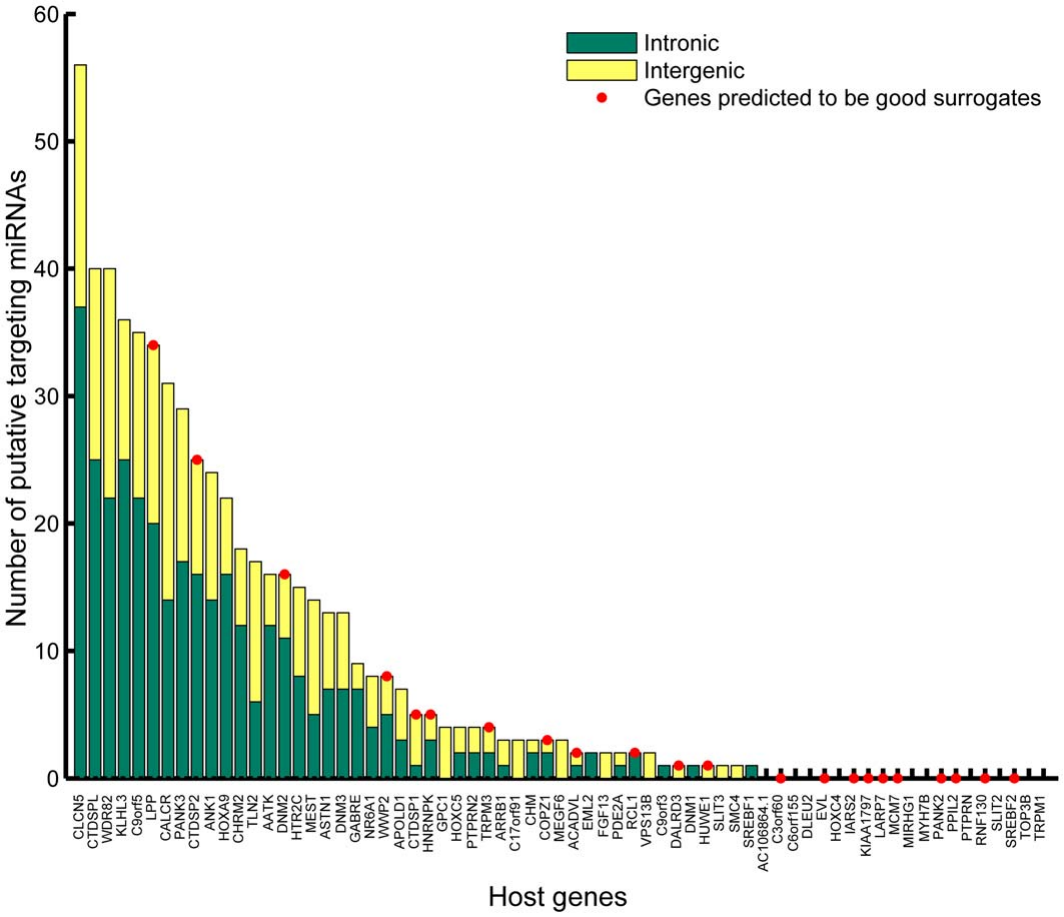
Number of intergenic and intronic miRNAs that putatively target our set of host genes. Bars marked by red circles are associated with the genes predicted to be good surrogates.

Even if a host gene and intronic miRNA are expressed from the same promoter, they could have different expression levels due to different post-transcriptional regulation. To investigate this, we examined the predicted miRNA targets within the 3′ UTRs of host genes. We found host genes are targeted by miRNAs much more than non-host genes (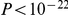, Wilcoxon ranksum test) though we were unable to detect a preference for targeting by intronic versus intergenic miRNAs (Fig S5). However, we found that negatively enriched host genes have significantly fewer (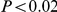, Wilcoxon ranksum test) miRNA targets than non-negatively enriched hosts (Fig. 10). So, down-regulation of the host gene by other miRNAs could provide another possible explanation for why some host expression levels are bad surrogates for those of their intronic miRNAs. The pattern of interactions among host genes and their intronic miRNAs suggests that there may be some hierarchical structure in intronic miRNA-based regulation (Fig S6).

### 7-Correlation measurements are not good indicators of surrogacy

**Figure 11:**
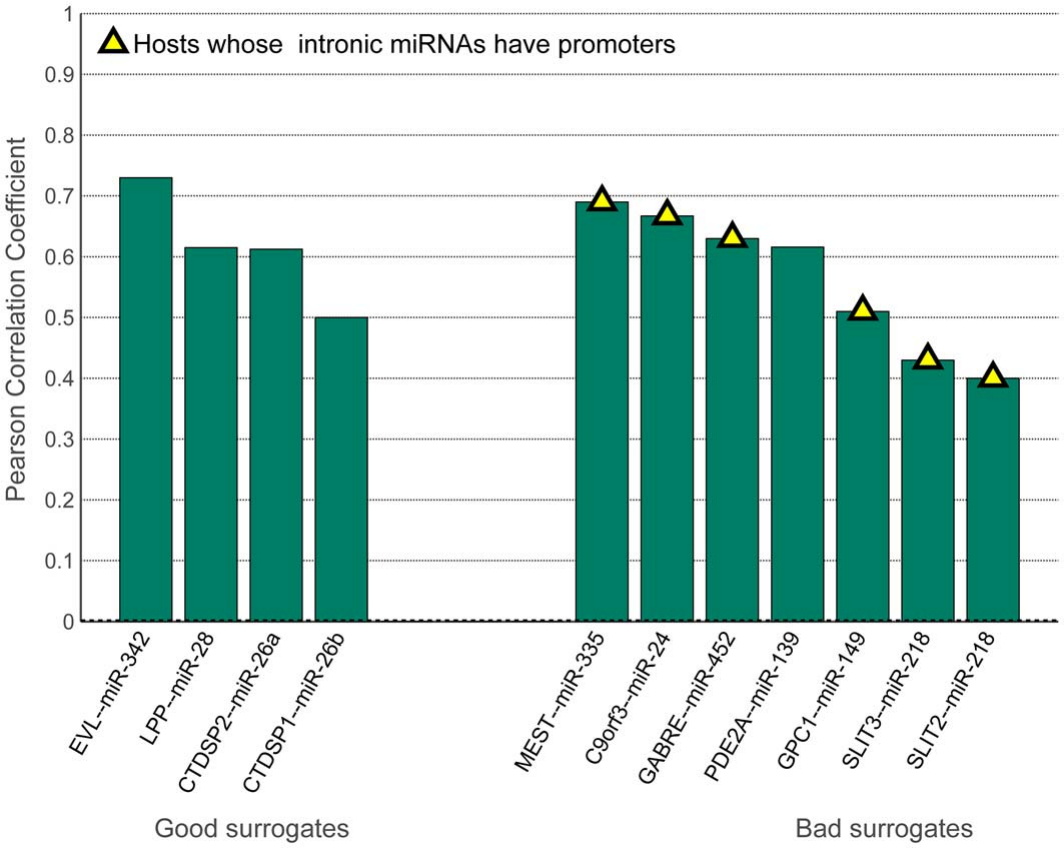
Pearson correlation coefficients averaged over five correlation datasets. (Table S6) Only those host-intronic miRNAs pairs which are significant (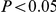) in at least two datasets and overlap with our host gene list are considered. The hosts marked with a yellow triangle contain intronic miRNAs with predicted independent promoters.

Correlation between the expression patterns of the host genes and their intronic miRNAs in a single dataset are not a good indicator of surrogacy. We observed that correlation measurements reported by five different groups are highly non-overlapped and somehow inconsistent (See File S1, Fig S7, Table S5). Only 11 host-miRNA pairs show high positive correlation (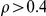) at least in two of these five datasets (Fig. 11). Out of these 11 host genes, 4 host genes are predicted to be good surrogates by our model. While the intronic miRNAs of none of these 4 hosts have promoters, 6 out of 7 hosts predicted to be bad surrogates have intronic miRNAs with promoters (Fig. 11). Thus, 7 highly correlated host-intronic miRNA pairs pass neither our criteria nor the promoterless condition.

## Discussion

**Figure 12:**
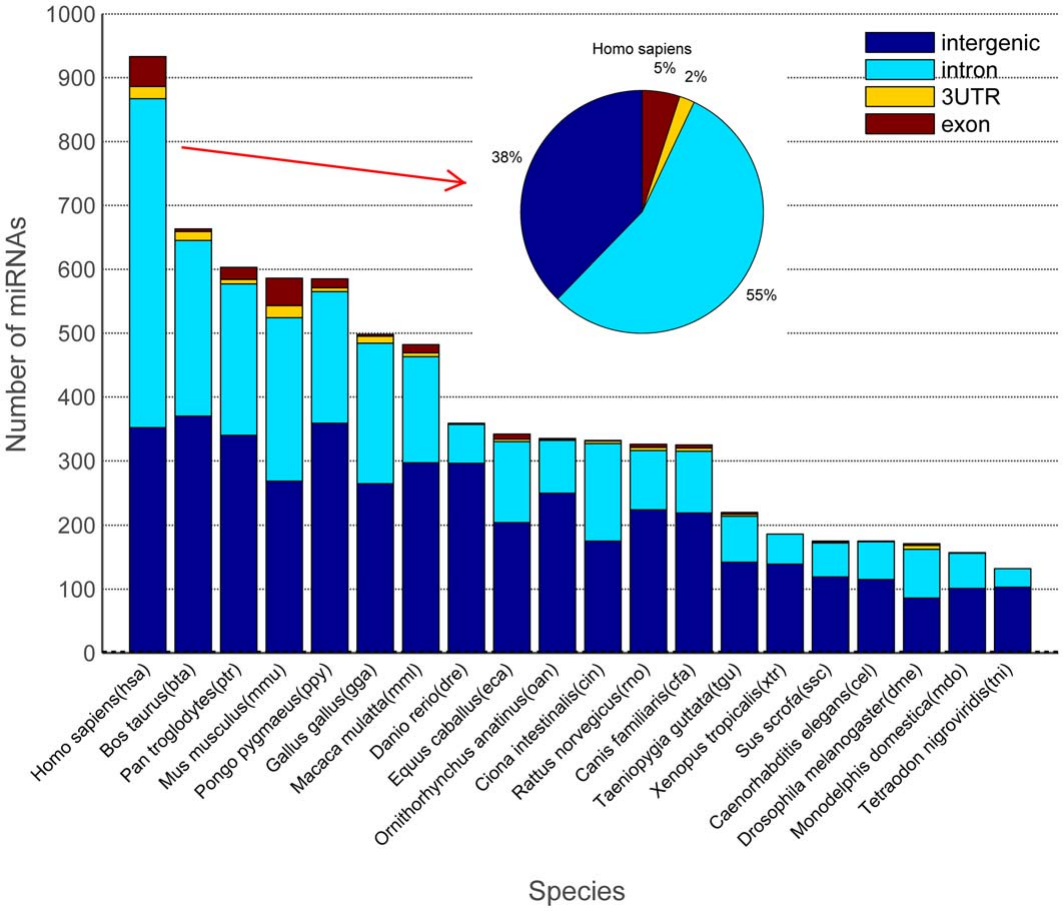
Intronic miRNAs comprises a significant portion of identified miRNAs in other species. Stack bars showing the number of miRNAs located in exon (brown), 3′UTR (yellow), intron (cyan), and intergenic regions (blue) in 20 species for which more than 100 microRNAs have been detected. Data are retrieved from miRBase (v.15).

InMiR models the combinatorial effect of miRNAs using a simple and biologically plausible linear model. Because we use ordinary linear regression for target prediction, InMiR is fast and easy to update to incorporate new mRNA expression data. We used data from 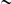
1,500 gene expression arrays to predict interactions in human between 57 intronic miRNAs and 3,864 potential targets. InMiR can also be readily applied to other species beside human because intronic miRNAs constitute a large portion of the miRNA complement of a variety of species (Fig. 12).

Unlike previously described methods, InMiR does not assume that all host genes have expression levels that are equally good surrogates. The set of host genes predicted by InMiR to be bad surrogates is enriched for those with predicted intronic promoters as well as having a larger number of microRNA target sites in their 3′ UTRs.

**Figure 13:**
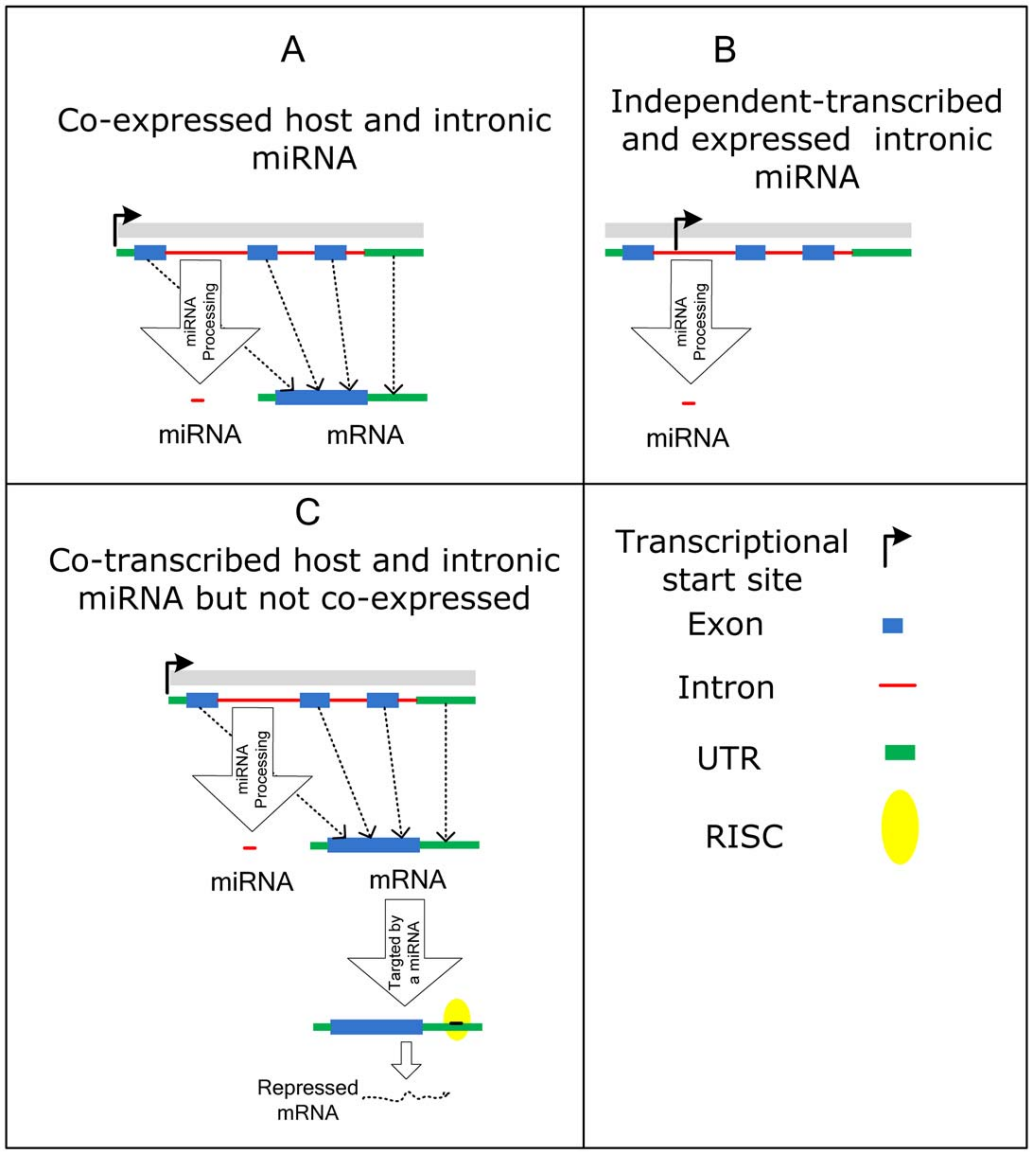
Regulatory mechanisms. Three possible scenarios for the transcription and expression of a host and its intronic miRNA.

As shown in Fig. 13, our observations suggest at least three types of regulatory relationships between host genes and their intronic microRNAs: (a) an intronic miRNA and its host gene are transcribed from the same promoter; the mature miRNA is then processed from intron before or after splicing using Drosha or independently (mirtrons) and the subsequent steady-state expression levels of the host and intronic miRNA are highly correlated (Fig6.a); (b) an intronic miRNA has its own promoter and is transcribed independently from the host gene at least some of the time (Fig 6.b); (c) the intronic miRNA and host are transcribed from the same promoter but the post-transcriptional regulation of the host gene expression levels is different than those of the miRNA (Fig 6.c). For example, a host gene could be down-regulated by its own intronic miRNA; we found three self-regulated hosts, all of which were predicted as bad surrogates by InmiR (Fig S8) or host genes could be down-regulated by other co-expressed miRNAs.

The host gene / intronic miRNA interactions that we observe suggest a variety of new regulatory mechanisms. For example, tightly coupled host gene and intronic miRNA expression could support a rapid “biological switch” in cellular state in which host gene expression also expresses an intronic miRNA that immediately down-regulates genes expressed in the competing state (Fig. S9).

Our observation raise a number of interesting questions. Are intronic miRNAs with their own promoter ever expressed from the host gene's promoter? How is this decision regulated? How does the independent transcription of an intronic miRNA affect host gene transcription? Does the processing of intronic miRNA interfere with splicing? This may depend on whether Drosha cleaves the pre-miRNA before or after splicing. Kim and Kim [56] speculated that both mechanisms may occur but no conclusive results can be drawn yet. Answers to these not well-understood mechanisms provide a clearer picture of intronic miRNA biogenesis.

## Materials and Methods

### 1-Microarray data

140 curated gene expression data sets, called GDS, were downloaded from Gene Expression Omnibus (GEO) using the MATLAB Bioinformatics toolbox function getgeodata.m. The list of these GDSs are given in Table S1. Each dataset is then processed as follows. First, we excluded those genes for which we have missing values. Then we filtered out genes with absolute values less than 10th percentile using MATLAB function genelowvalfilter.m. The expression profile related to the host gens are normalized so that all have length one. Mathematically this means 
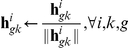
. For the target genes, we obtain the decrease in expression level as 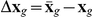 where 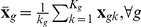.

### 2-Maximum Likelihood Estimation

The maximum likelihood estimate of 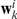 is given by
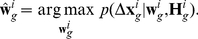
(6)The vector 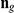 is modeled by a zero mean white Gaussian noise of the form
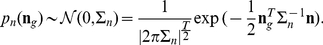
(7)If we assume that the noise process has a diagonal covariance matrix of the form 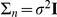 where 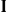 denotes the identity matrix, then maximum likelihood function is given by
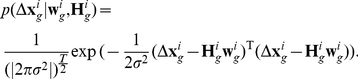
(8)Thus, maximizing the log of 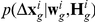 is equivalent
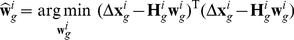
(9)


### 3-Predicting miRNA targets using inverse correlation (CORR method)

Gennarino and colleague [16] recently described an algorithm, HOCTAR, that predict intronic microRNA targets based on inverse correlation of their host genes with other mRNAs across a large number of datasets. As we have previously demonstrated [71], linear models that consider the impact of multiple potential miRNA regulators generate more accurate target predictions than simple correlations, consistent with recent observations of miRNA-target interactions [31], [32]. To assess whether these observations hold for target predictions based on host gene expression, we also assessed a version of our method in which we replace the weights with correlations. The resulting algorithm is very similar to HOCTAR.

In particular, we denote the correlation coefficient by 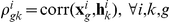 where 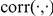 represents the Pearson correlation coefficient. We then use these correlations 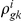 for real and permuted datasets in the place of weights to calculate the P-value based enrichment measures as described in Section II.C. We call this method as CORR.

### 4-Processing hosts and targets data

We retrieved the mirRBase gene context repository and extracted all human intronic miRNA-host gene association (Table S2). We also downloaded 140 gene expression datasets (GDS) from Gene Expression Omnibus (GEO) which were built on the Affymetrix HG-U133 microarray platform [16] using MATLAB function *getgeodata.m* (Table S1 and materials). Only those probe IDs that could be mapped to gene symbols (according to HGNC) were considered for analysis. We used the list of putatively predicted target genes (9448) and their intronic miRNAs (134) from the TargetScan (release 5.1) repository.

### 5-Pseudo code for implementing InmiR

**for** 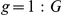
(number of target genes)

 ▸find all intronic miRNAs which putatively target 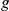 using    TargetScan

 ▸map intronic miRNAs to their host genes ,
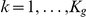


 **for** 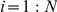
(number gene expression datasets)

  ▸extract the expression data of the host genes, 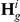


  ▸extract the expression data of the target gene, 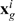


  ▸solve 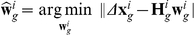


  ▸permute the rows using a permuted matrix, 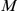, to get 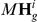


  ▸solve 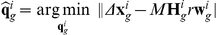


 **end**

 **for** 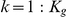


  ▸compute the P-values:

  
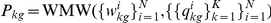


  
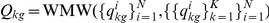


 **end**

**end**

▸set two classes of data I: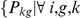} and II:
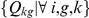


▸plot ROC curve and determine a cutoff point (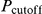) to get   almost zero false positive

▸declare the interaction between host gene 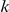 and target gene 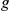   significant if 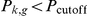


## Supporting Information

Figure S1 **The cumulative distribution function obtained from ULM.** The cumulative distribution functions of the negative 10 based logarithm of the p-values for the actual and permuted host-target interactions obtained form ULM (dashed and solid blue lines), and CORR (dashed and solid red lines). The cutoff point was set to 2 (the dashed black vertical line) and all p-values beyond this point are declared significant.
(TIF)

Figure S2
**The cumulative distribution function obtained from CLM.** The cumulative distribution functions of the negative 10 based logarithm of the p-values for the actual and permuted host-target interactions obtained form constrained linear model (CLM)–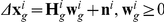
–(dashed and solid blue lines), and ULM.(TIF)

Figure S3
**Receiver Operating Characteristic (ROC) curve analysis for ULM and CLM.**Receiver Operating Characteristic (ROC) curve analysis to determine the cutoff point. We set the cutoff point to 0.01 (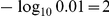) to identify significant host-target interactions. The blue and green curves show the ROC associated with ULM and CLM.(TIF)

Figure S4
**The weights CDFs and p-values obtained from ULM.** Plots e-f: the CDFs of the weights 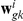 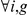 for seven host genes obtained from constrained linear model (CLM)–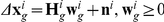
– with the actual (e) and permutation data (f). The thick gray line in each plot is the CDF obtained from the pooled permutation data for each method. Table lists the 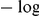 p-values (Willcoxon ranksum test) showing the probability that the weight or correlation data are drawn from the pooled permutated data (see (4) and (5) for detail). It should be noted that the host gene MIRHG1 was excluded for analysis since the expression data related this host gene did not exist in the retrieved dataset.(TIF)

Figure S5 **The CDFs of the number of miRNAs targeting host and non-host genes.** Top: the cumulative distribution of the number of miRNAs targeting host (blue) and non-host genes (red). The inset shows the CDF of 3′ UTR length of hosts(bule) and non-host genes (bule). Bottom: the CDF of the number of miRNAs targeting host (blue) and non-host genes (red) per base; that is, number of target /3′UTR length. The CDFs are obtained from analyzing 367 host genes and 17000 non-host genes.
(TIF)

Figure S6 **Host genes targeted by intronic miRNAs of other hosts.** Host genes targeted by intronic miRNAs of other hosts. The nodes corresponding to hosts predicted to be good surrogates are shown in red.
(TIF)

Figure S7 **Scatter plots of five correlation datasets.** Scatter plots of five correlation datasets (Table S4). (a) the scatter plot of Rad's data versus Liang's, Wang's, Ruike's, and Baskerville's data. (b) the scatter plot of Liang's data versus Wang's, Ruike's, and Baskerville's data. (c) the scatter plot of Wang's data versus Ruike's and Baskerville's data. (d) the scatter plot of Ruike's data versus Baskerville's data.
(TIF)

Figure S8 **The host genes targeted by their own intronic miRNAs.** The host genes in our dataset which are targeted by their own intronic miRNAs. All of these hosts are predicted to be bad surrogates.
(TIF)

Figure S9 **Host and intronic miRNA resemble a “biological switch”.** Tightly coupled host gene and intronic miRNA expression could support a rapid “biological switch” in cellular state in which host gene expression also expresses an intronic miRNA that immediately down-regulates genes expressed in the competing state.
(TIF)

Table S1 List of GDS data for analysis. The identifiers of Gene Datasets (GDS) retrieved from the Gene Expression Omnibus repository.
(XLS)

Table S2 The excel file contains all intronic-host genes pairs. Data are retrieved from MirBase v.15.
(XLS)

Table S3 The excel file, consisting of 6 sheets, contains the entire p-values obtained from interactions between 3864 intronic miRNAs targeted genes and 57 hosts genes using the CLM, ULM, and CORR methods. sheet 1 p-values from the CLM model. sheet 2 p-values from the CLM model with permuted data. sheet 3 p-values from the ULM model. sheet 4 p-values from the ULM model with permuted data. sheet 5 p-values from the CORR model. sheet 6 p-values from the CORR model with permuted data. The names of the targeted genes and host genes are given in the first row and column of the first sheet. Note that a zero in (*i,j*) in the tables shows that the *i*th gene is not a target of the intronic miRNAs of the *j*th host.
(XLS)

Table S4The excel file contains all target-intronic miRNA pairs and their scores. column one: target genes. column two: intronic mirnas. column three: host genes. column four: scores (pvalues)– scores 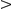
2 are significant. column five flag = 1 negative and flag = 1 positive interactions.(XLS)

Table S5 coefficients. Correlation coefficients obtained from five different datasets, namely Baskerville et al., Liang et al., Wang et al., Ruike et al. , and Rad. The data reported by Wang et al. are in terms of p-values. A empty cell in the table shows that either the data was not available for the host-intronic miRNA pair or the correlation coefficient was negative or insignificant.
(XLS)

File S1 Host-intronic mirnas correlation data.
(PDF)
