# Supplementary figures and images for: Computational Prediction of Intronic microRNA Targets using Host Gene Expression Reveals Novel Regulatory Mechanisms
Source: PLoS One. 2011 Jun 9;6(6):e19312. doi: 10.1371/journal.pone.0019312 (PMC3111417; doi:10.1371/journal.pone.0019312)

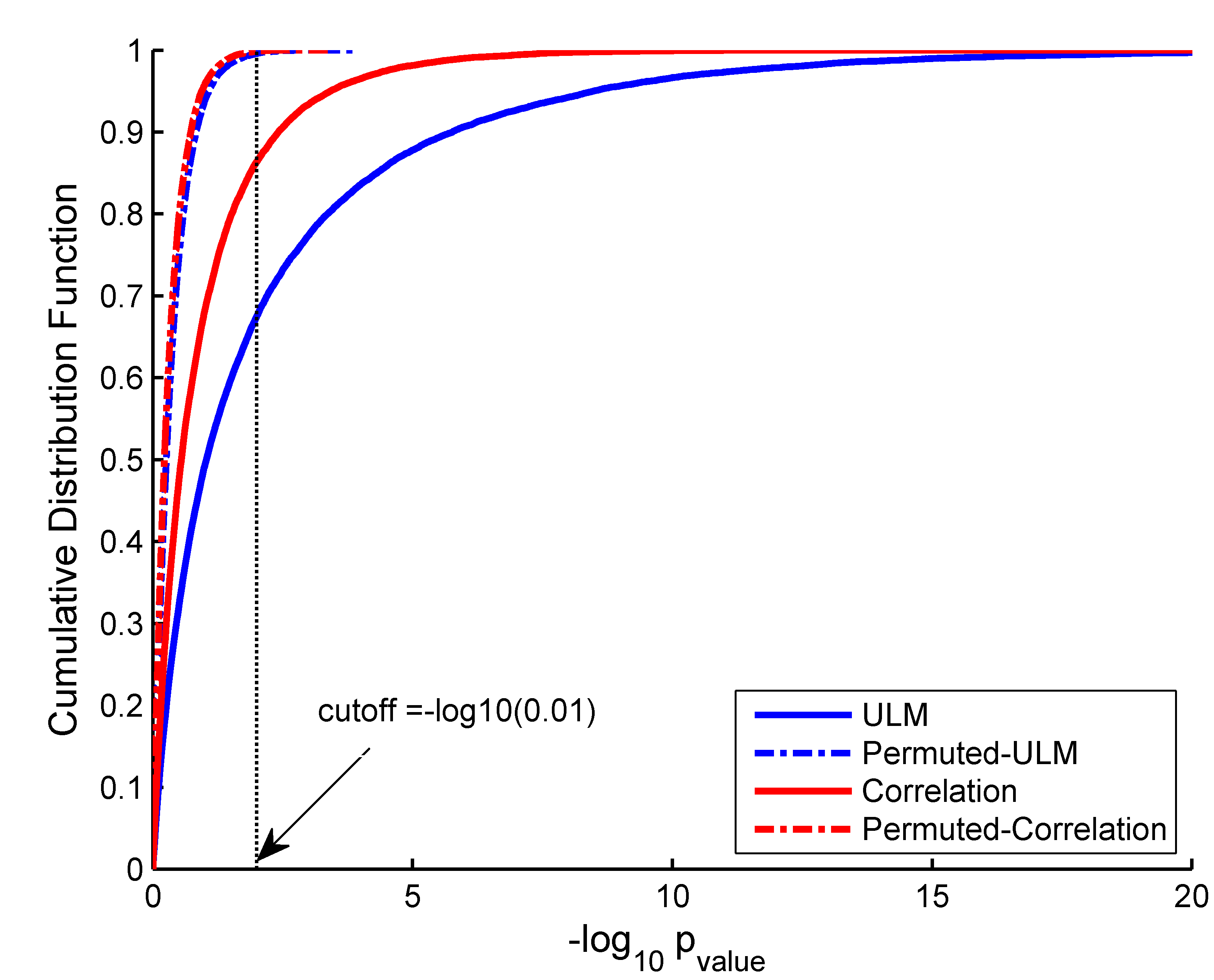

Supplement: Figure S1 — The cumulative distribution function obtained from ULM. The cumulative distribution functions of the negative 10 based logarithm of the p-values for the actual and permuted host-target interactions obtained form ULM (dashed and solid blue lines), and CORR (dashed and solid red lines). The cutoff point was set to 2 (the dashed black vertical line) and all p-values beyond this point are declared significant. (TIF) [file pone.0019312.s001.tif]

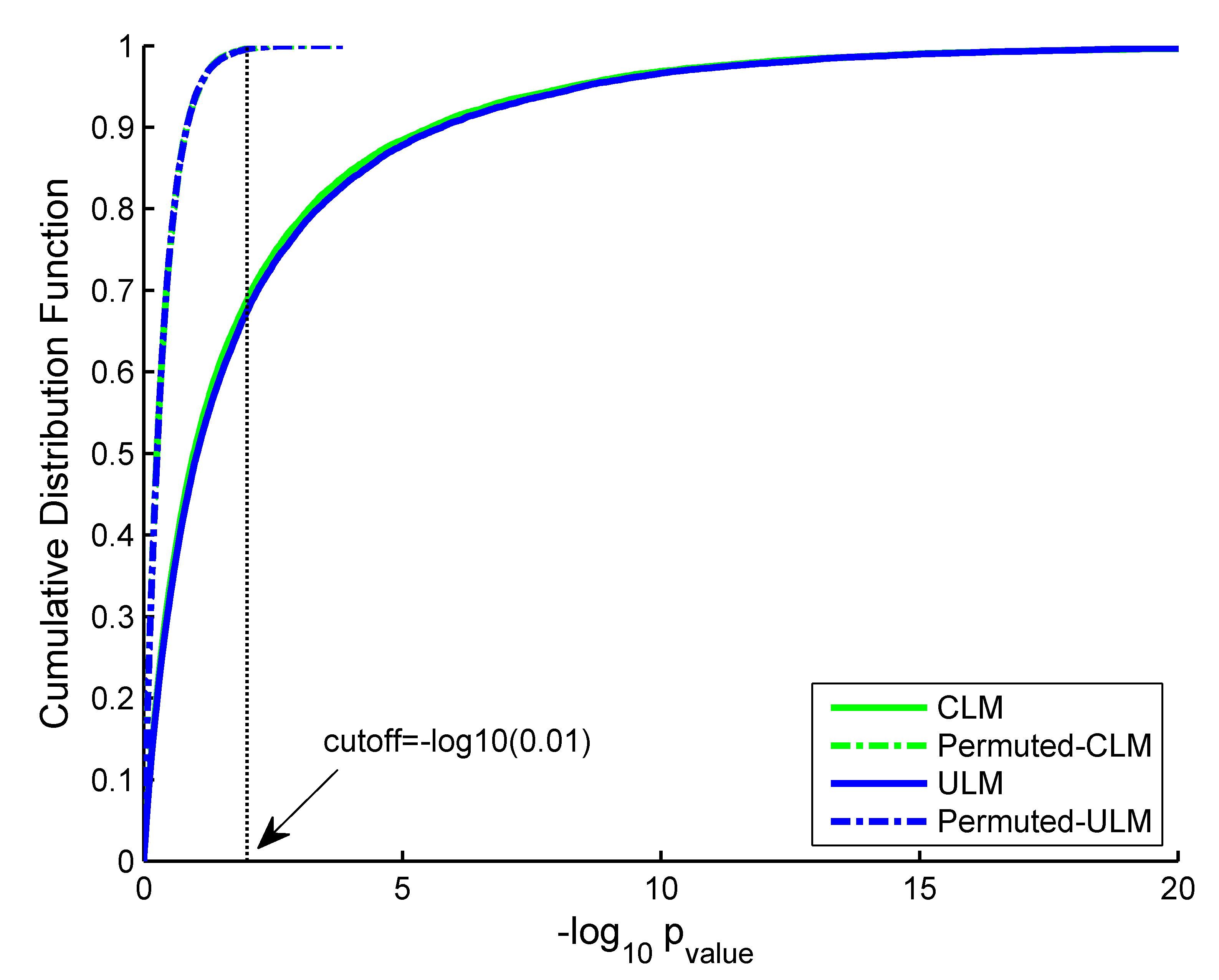

Supplement: Figure S2 — The cumulative distribution function obtained from CLM. The cumulative distribution functions of the negative 10 based logarithm of the p-values for the actual and permuted host-target interactions obtained form constrained linear model (CLM)––(dashed and solid blue lines), and ULM. (TIF) [file pone.0019312.s002.tif]

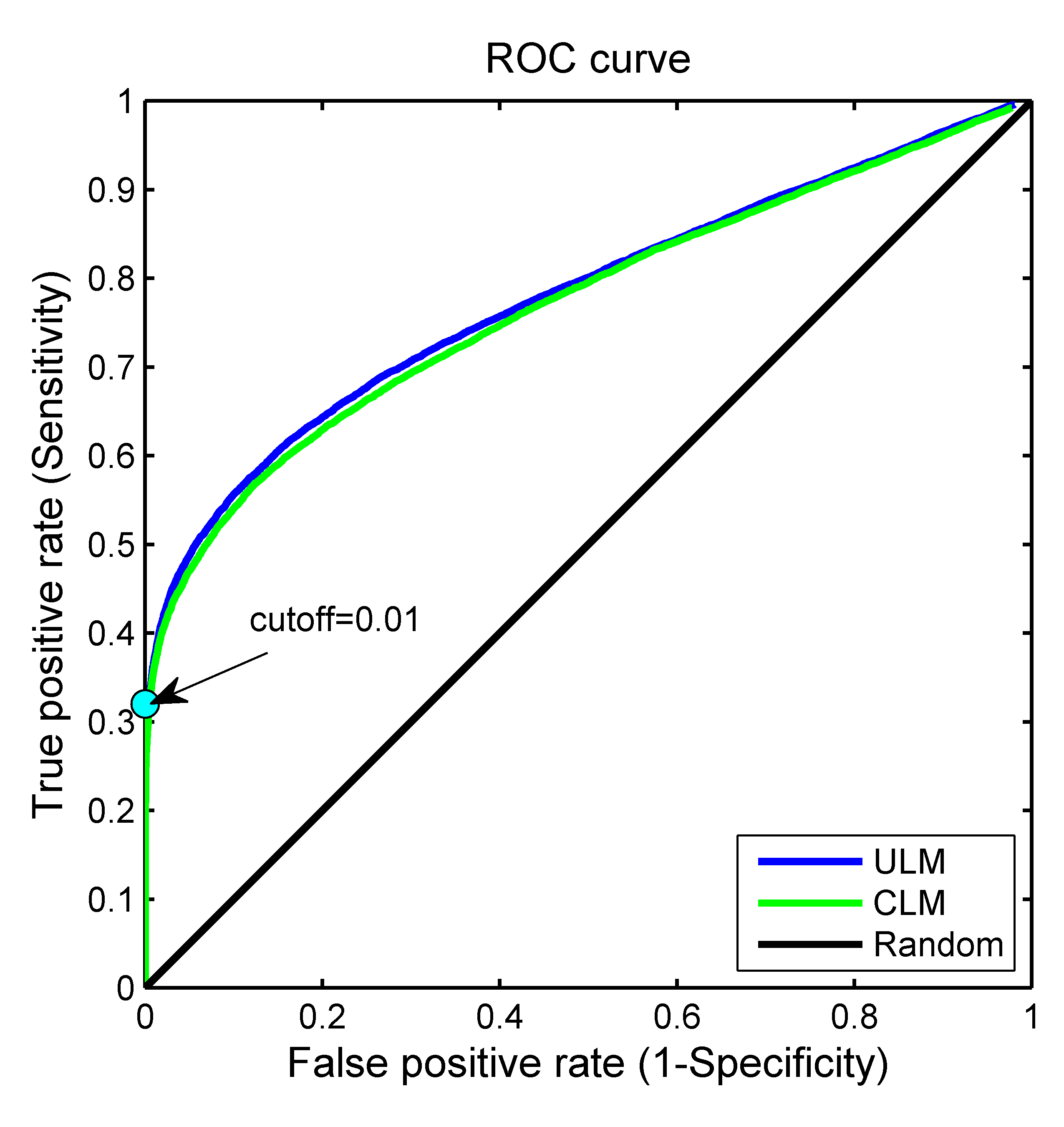

Supplement: Figure S3 — Receiver Operating Characteristic (ROC) curve analysis for ULM and CLM.Receiver Operating Characteristic (ROC) curve analysis to determine the cutoff point. We set the cutoff point to 0.01 () to identify significant host-target interactions. The blue and green curves show the ROC associated with ULM and CLM. (TIF) [file pone.0019312.s003.tif]

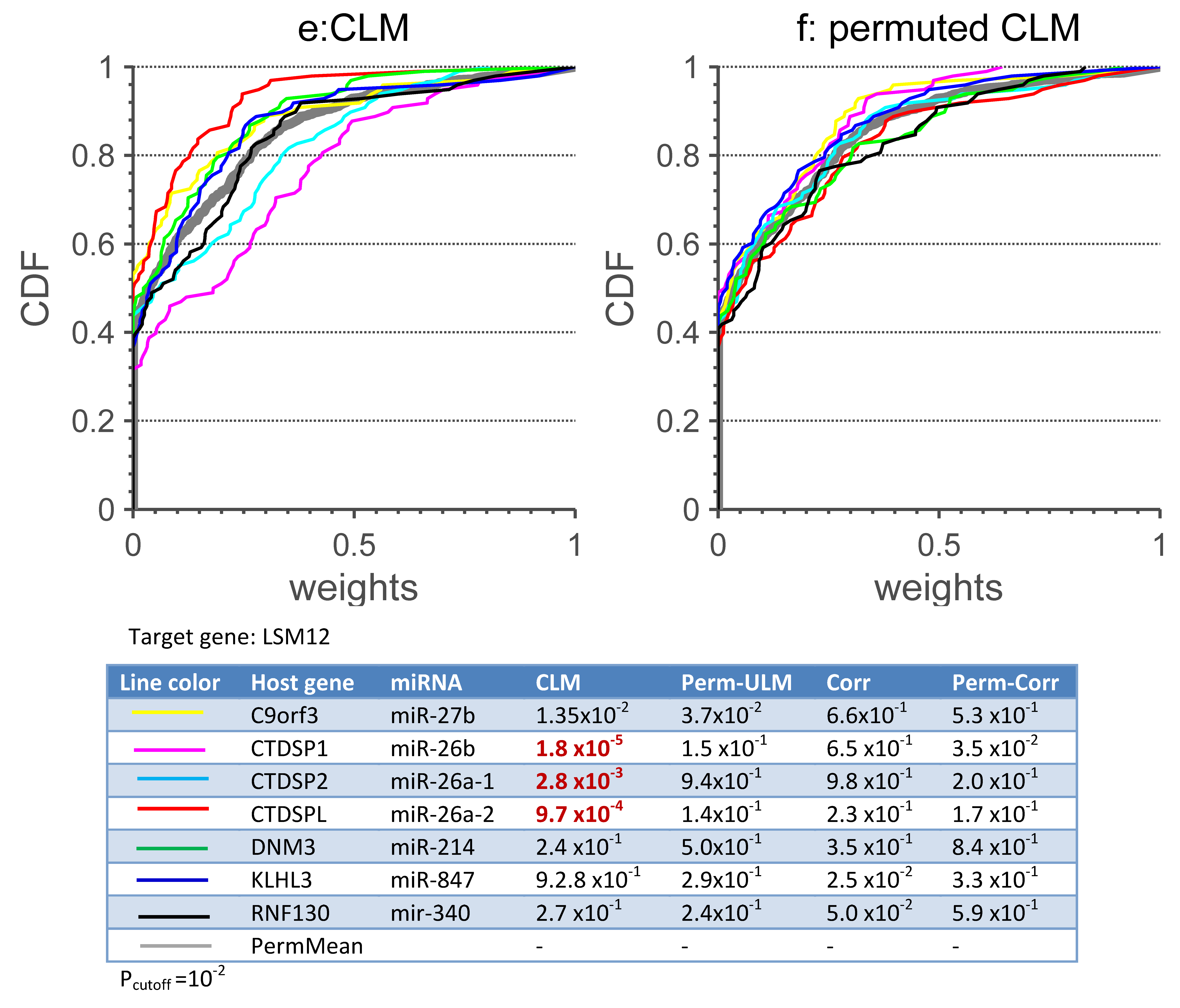

Supplement: Figure S4 — The weights CDFs and p-values obtained from ULM. Plots e-f: the CDFs of the weights for seven host genes obtained from constrained linear model (CLM)–– with the actual (e) and permutation data (f). The thick gray line in each plot is the CDF obtained from the pooled permutation data for each method. Table lists the p-values (Willcoxon ranksum test) showing the probability that the weight or correlation data are drawn from the pooled permutated data (see (4) and (5) for detail). It should be noted that the host gene MIRHG1 was excluded for analysis since the expression data related this host gene did not exist in the retrieved dataset. (TIF) [file pone.0019312.s004.tif]

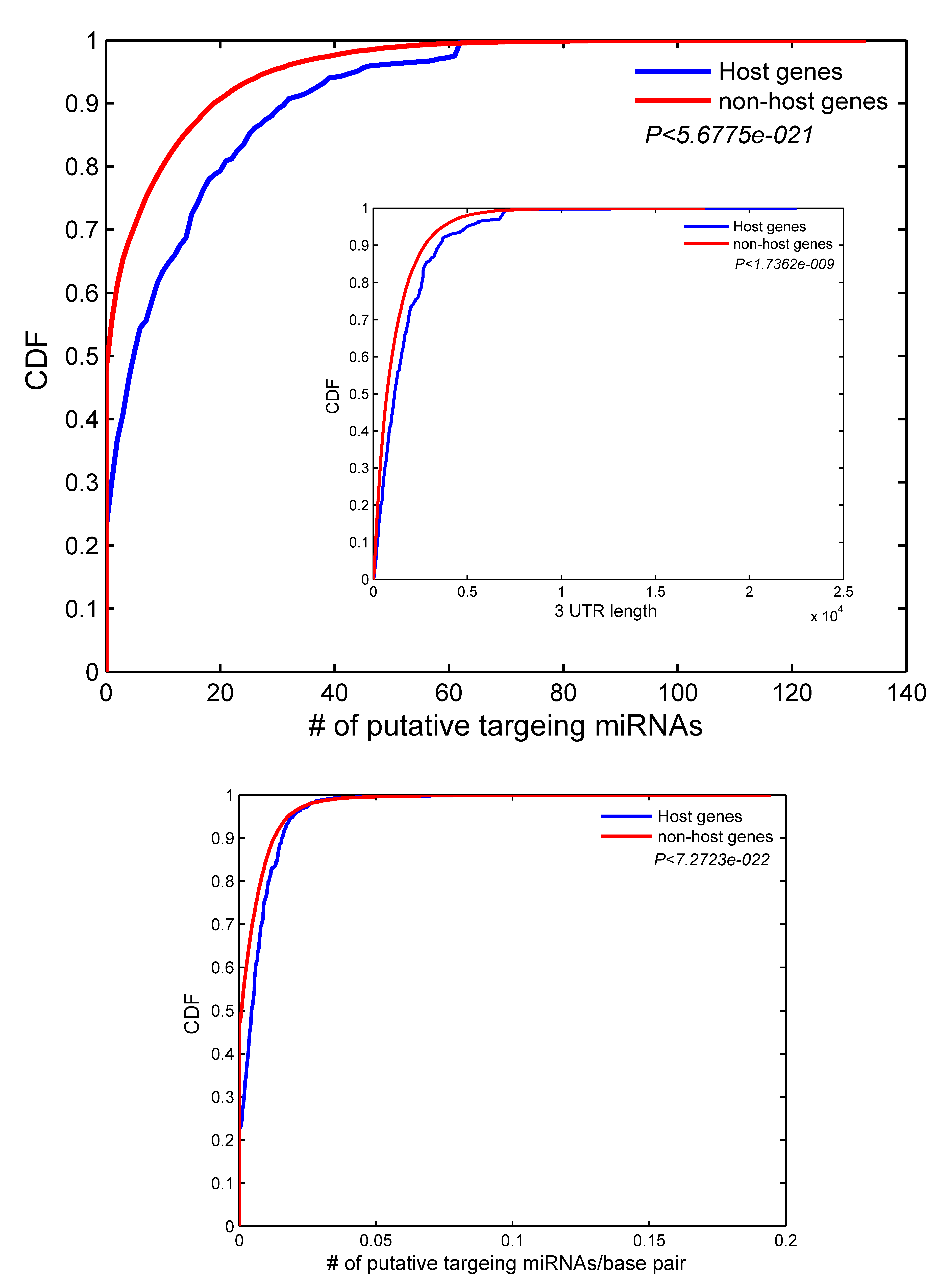

Supplement: Figure S5 — The CDFs of the number of miRNAs targeting host and non-host genes. Top: the cumulative distribution of the number of miRNAs targeting host (blue) and non-host genes (red). The inset shows the CDF of 3′ UTR length of hosts(bule) and non-host genes (bule). Bottom: the CDF of the number of miRNAs targeting host (blue) and non-host genes (red) per base; that is, number of target /3′UTR length. The CDFs are obtained from analyzing 367 host genes and 17000 non-host genes. (TIF) [file pone.0019312.s005.tif]

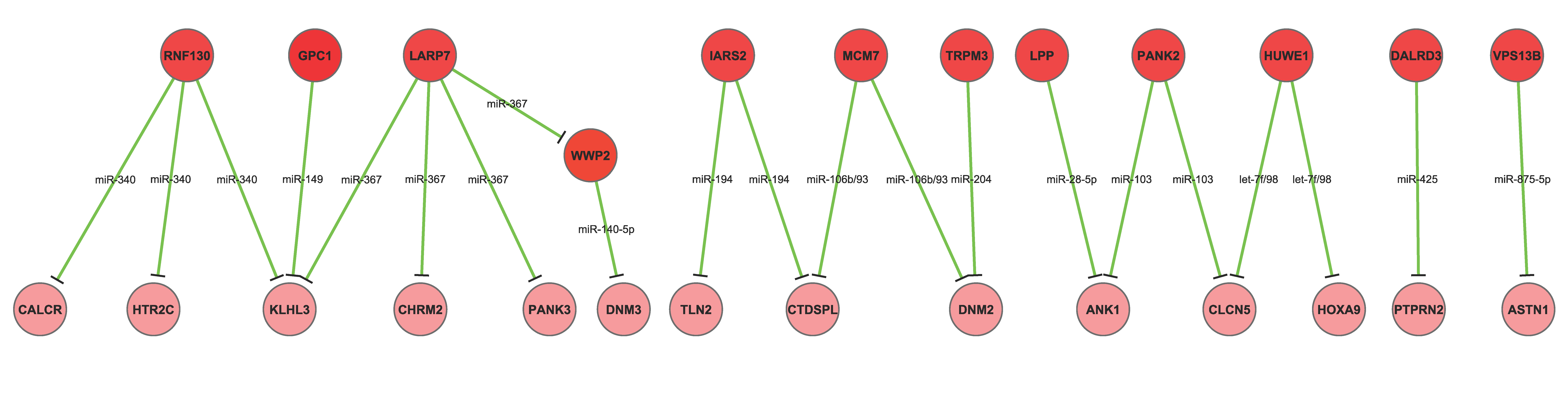

Supplement: Figure S6 — Host genes targeted by intronic miRNAs of other hosts. Host genes targeted by intronic miRNAs of other hosts. The nodes corresponding to hosts predicted to be good surrogates are shown in red. (TIF) [file pone.0019312.s006.tif]

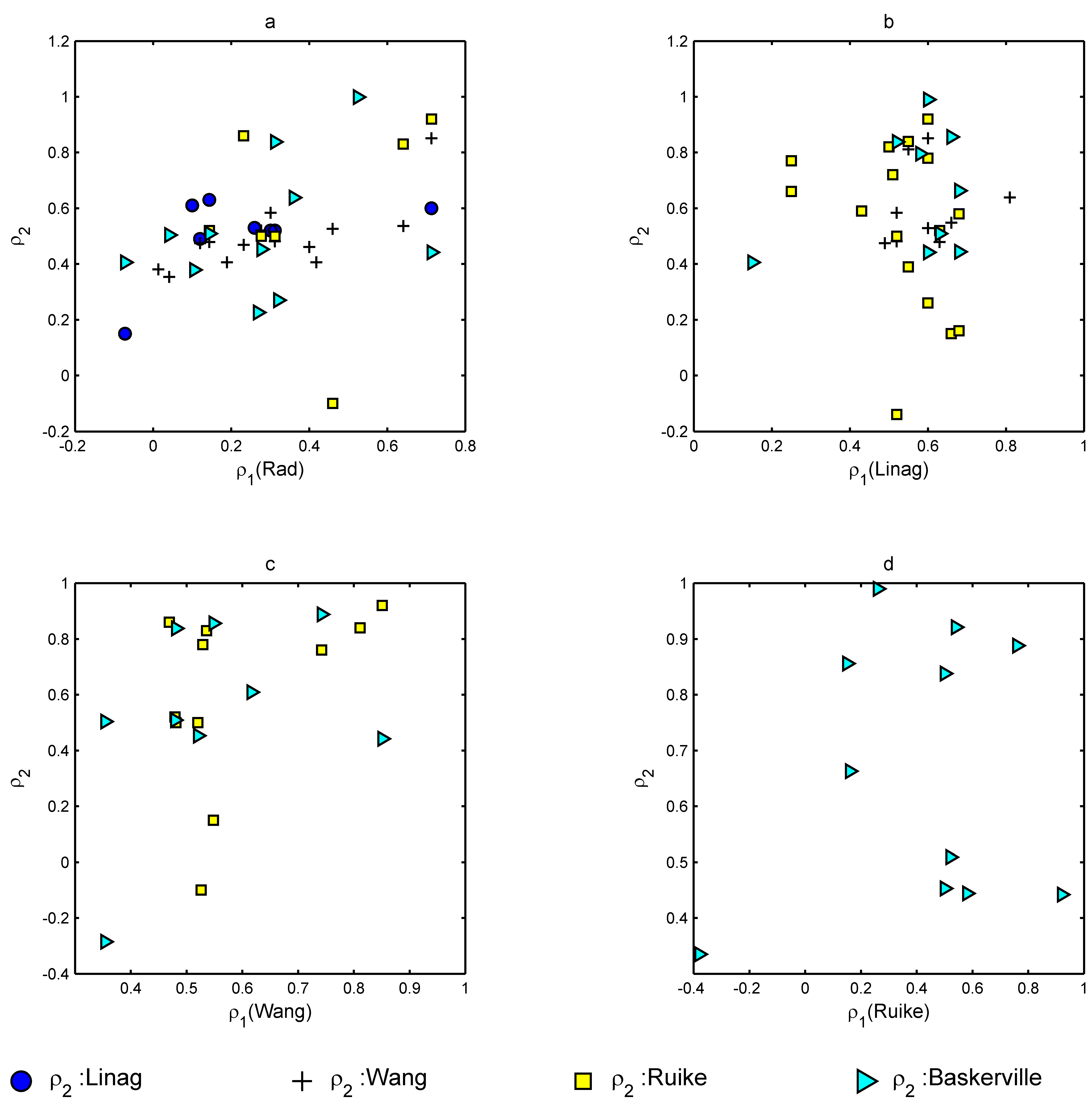

Supplement: Figure S7 — Scatter plots of five correlation datasets. Scatter plots of five correlation datasets (Table S4). (a) the scatter plot of Rad's data versus Liang's, Wang's, Ruike's, and Baskerville's data. (b) the scatter plot of Liang's data versus Wang's, Ruike's, and Baskerville's data. (c) the scatter plot of Wang's data versus Ruike's and Baskerville's data. (d) the scatter plot of Ruike's data versus Baskerville's data. (TIF) [file pone.0019312.s007.tif]

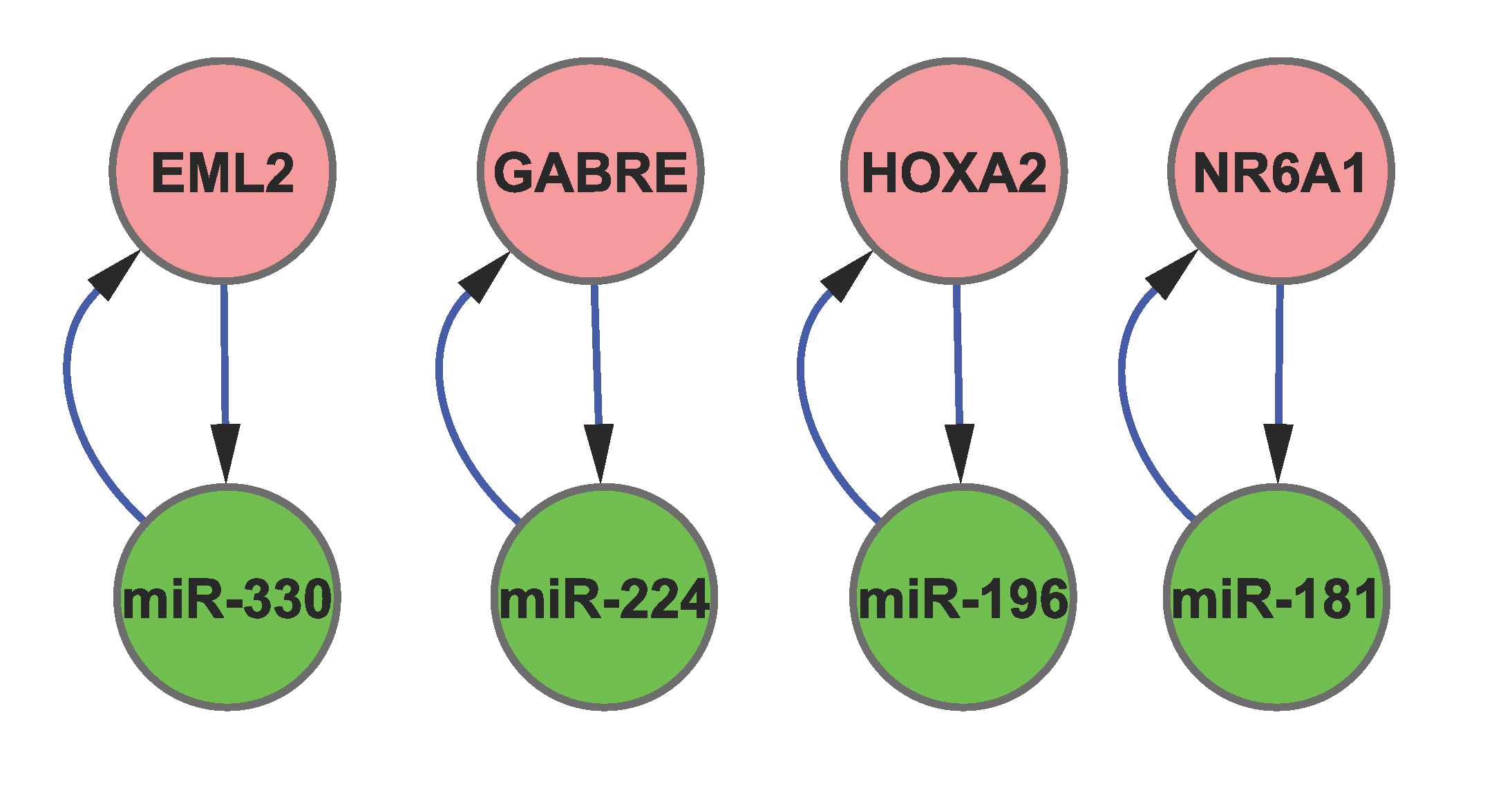

Supplement: Figure S8 — The host genes targeted by their own intronic miRNAs. The host genes in our dataset which are targeted by their own intronic miRNAs. All of these hosts are predicted to be bad surrogates. (TIF) [file pone.0019312.s008.tif]

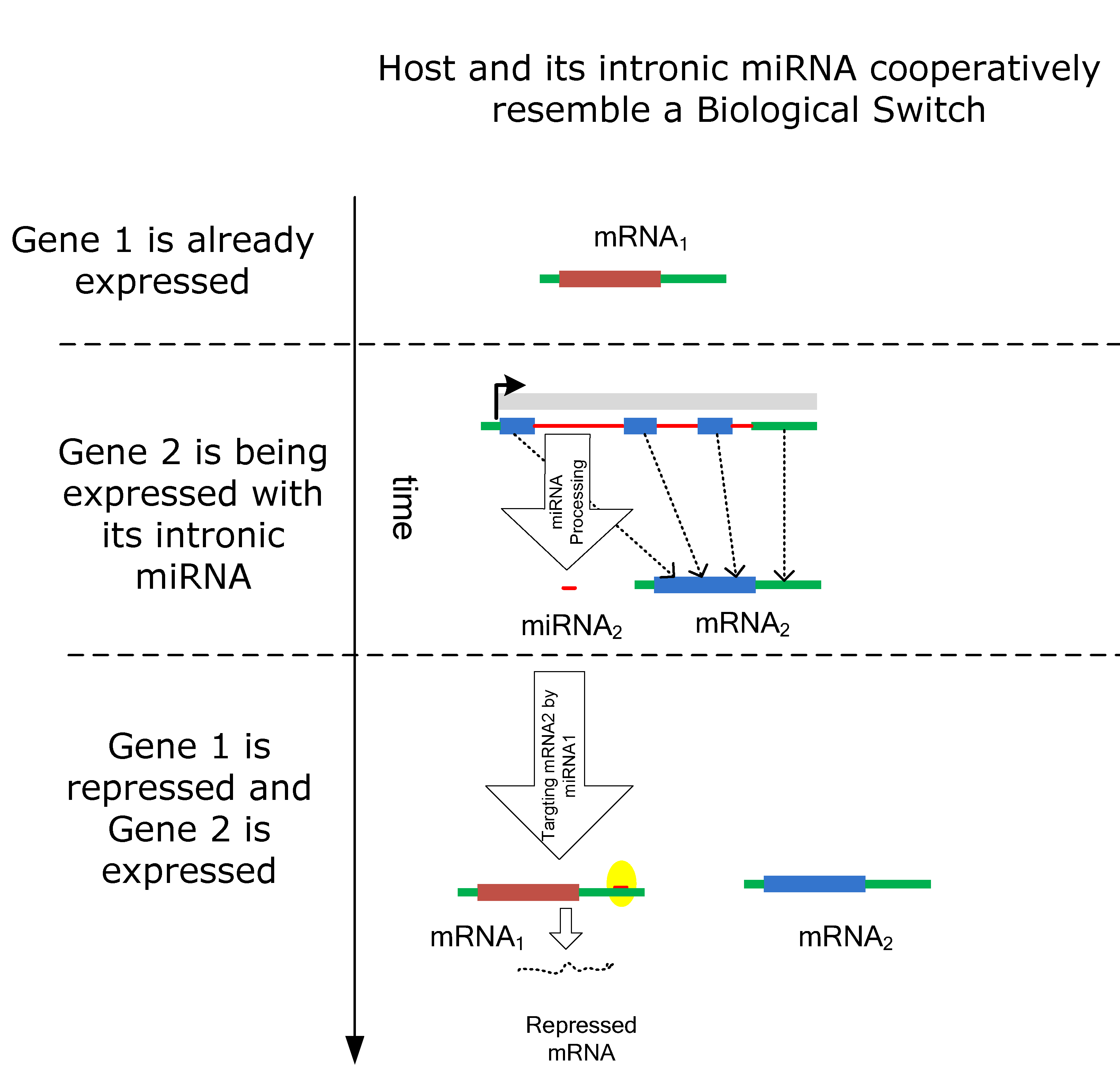

Supplement: Figure S9 — Host and intronic miRNA resemble a “biological switch”. Tightly coupled host gene and intronic miRNA expression could support a rapid “biological switch” in cellular state in which host gene expression also expresses an intronic miRNA that immediately down-regulates genes expressed in the competing state. (TIF) [file pone.0019312.s009.tif]
