# Supplementary material for: Computational Prediction of Intronic microRNA Targets using Host Gene Expression Reveals Novel Regulatory Mechanisms
Source: PLoS One. 2011 Jun 9;6(6):e19312. doi: 10.1371/journal.pone.0019312 (PMC3111417; doi:10.1371/journal.pone.0019312)
Supplement: File S1 — Host-intronic mirnas correlation data. (PDF) [file pone.0019312.s015.pdf]

Supplementary Materials

Computational Prediction of Intronic miRNA Targets Using Host Gene Expression Reveals Novel Regulatory Mechanisms

M.H.Radfar, W. Wong, and Q Morris

I. HOST-INTRONIC MIRNAS CORRELATION DATA

We collected the correlation results reported by Wang et al. [1], Liang et al. [2], Baskerville et al. [3], and Ruike et al. [4]. Wang's data, reported in terms of p-values, are transformed to Pearson correlation coefficients to be consistent with other data. The transformation is done based on the significance of a correlation coefficient test [5]. In addition, we applied the data given in [6] and [7] and computed the correlation between the matched host genes and intronic miRNAs;we refer this method as Rad. In order to compute the correlation between the expression profiles of miRNAs and mRNAs in Rad data, we analyzed the data collected in [8]. Ritchie et al. analyzed miRNAs expression data cloned by Landgraf, et al. [7]. After downloading their data and processing them we obtain the expressions of 117 human miRNAs and the expression of 22283 genes over 117 samples. We then computed the correlation between all miRNA-mRNA pairs.

In this way, we obtain correlation coefficients for 84 host-intronic miRNA pairs from five different datasets (Table S5). We expect that co-expressed host-miRNA pairs show strong correlation in at least two of these five datasets. The scatter plots (Fig. **??**) of the correlation data however show that the data are highly non-overlapping and somehow inconsistent, suggesting that solely relying on correlation data may not be sufficient to declare a host-intronic miRNA pair co-transcribed.

REFERENCES

[1] W. Yu-Ping and L. Kuo-Bin, "Correlation of expression profiles between microRNAs and mRNA targets using NCI-60 data," *BMC Genomics*, vol. 10.

[2] Y. Liang, D. Ridzon, L. Wong, and C. Chen, "Characterization of microRNA expression profiles in normal human tissues," *BMC genomics*, vol. 8, no. 1, pp. 166, 2007.

[3] S. Baskerville and D. P. Bartel, "Microarray profiling of microRNAs reveals frequent coexpression with neighboring miRNAs and host genes," *RNA*, vol. 11, no. 3, pp. 241–247, 2005.

[4] Y. Ruike, A. Ichimura, S. Tsuchiya, K. Shimizu, R. Kunimoto, Y. Okuno, and G. Tsujimoto, "Global correlation analysis for micro-RNA and mRNA expression profiles in human cell lines," *Journal of human genetics*, vol. 53, no. 6, pp. 515–523, 2008.

[5] R. Lowry, "Concepts and applications of inferential statistics," *VassarStats: Web Site for Statistical Computation*, 2005.

[6] B. John, C. Sander, D.S. Marks, et al., "Prediction of human microRNA targets," *METHODS IN MOLECULAR BIOLOGY-CLIFTON THEN TOTOWA-*, vol. 342, pp. 101, 2006.

[7] P. Landgraf, M. Rusu, R. Sheridan, A. Sewer, N. Iovino, A. Aravin, S. Pfeffer, A. Rice, A.O. Kamphorst, M. Landthaler, et al., "A mammalian microRNA expression atlas based on small RNA library sequencing," *Cell*, vol. 129, no. 7, pp. 1401–1414, 2007.

[8] W. Ritchie, S. Flamant, and J.E.J. Rasko, "Predicting microRNA targets and functions: traps for the unwary," *Nature Methods*, vol. 6, no. 6, pp. 397–398, 2009.
